# Neuroimmune mechanisms in patients with atopic dermatitis during chronic stress

**DOI:** 10.1111/j.1468-3083.2007.02202.x

**Published:** 2008-01

**Authors:** SB Lonne-Rahm, H Rickberg, H El-Nour, P Mårin, EC Azmitia, K Nordlind

**Affiliations:** † Unit of Dermatology and Venereology, Department of Medicine, Karolinska University Hospital Solna, Stockholm, Sweden; ‡ Department of Medicine, Östra sjukhuset Gothenburg, Sweden; § Department of Biology and Psychiatry, New York University New York, NY, USA

**Keywords:** atopic dermatitis, innervation, mastcells, neuropeptides, receptors, serotonin, stress

## Abstract

**Objective:**

To identify pathoaetiological neuroimmune mechanisms in patients with atopic dermatitis (AD) and chronic stress, focusing at nerve density, sensory neuropeptides, and the serotonergic system.

**Methods:**

Eleven patients with AD with histories of stress worsening were included. Biopsies from involved and non-involved skin were processed for immunohistochemistry. Salivary cortisol test was done as a marker for chronic stress.

**Results:**

There were more acanthosis and fewer nerve fibres in epidermis and papillary dermis of involved compared with non-involved skin. Whereas there was no significant change in the number of substance P and calcitonin gene-related peptide–positive nerve fibres between the involved and non-involved skin, there was an increase in the epidermal fraction of 5-hydroxtrytamine 1A (5-HT1A) receptor and serotonin transporter protein (SERT) immunoreactivity in the involved skin. The number of 5-HT2AR, CD3-positive cells, and SERT-positive cells, most of them being CD3 positive, was increased in involved skin. There was an increase in mast cells in the involved skin, and these cells were often located close to the basement membrane. There was a strong tendency to a correlation between 5-HT2AR positive cells in the papillary dermis of involved skin and low cortisol ratios, being an indicator of chronic stress.

**Conclusion:**

A changed innervation and modulation of the serotonergic system are indicated in chronic atopic eczema also during chronic stress.

## Introduction

Atopic dermatitis (AD) is a common chronic skin disease, which affects about 2% to 3% of the adult population.^1^ It produces an extremely itchy skin. A marked increase in the prevalence of AD in urbanized societies has occurred during the past decades.^2^

The aetiology of AD is unknown but is probably multifactorial, with interactions between several genetic and environmental factors.^3^ Stress aggravates the symptoms.^4^

The skin controls and transmits contacts with the external world. It is an integral component of the immune, nervous, and endocrine systems, and there are numerous lines of crosstalk between these systems established intracutaneously.^5^ The skin contains an extensive neural network represented by unmyelinated sensory fibres and receptors for neuropeptides and neurotransmitters identical to those expressed in the central neuroendocrine system.^6^

The free nerve endings of the sensory nerves, C-fibres, are widely distributed in the epidermis, and most of them reach the stratum granulosum. Activation of sensory unmyelinated neurones evokes the release of neuropeptides, such as substance P.

The neuropeptides substance P and calcitonin gene-related peptide (CGRP) have specific biological effects and are a link between the neuroendocrine and immune axis.^7^, ^8^ Functional dysregulation of neuropeptides may be involved in the pathogenesis of AD.^9^, ^10^

The substance P–related receptor (R), the neurokinin-1 (NK-1) R, is expressed by human keratinocytes, endothelial cells, fibroblasts, and mast cells. Activation of this receptor stimulates proliferation of keratinocytes, fibroblasts, and endothelial cells.^8^, ^11^

C-fibres in the skin interact with mast cells. An increased number of mast cell nerve contacts are observed in dermal skin of AD compared with those in healthy skin.^12^

Serotonin [5-hydroxtrytamine (5-HT)] is an amine that acts as a neurotransmitter in a wide variety of sites in the central and peripheral nervous system.^13^ It is involved in numerous body functions. Functional dysregulation may lead to sleep disorders, anxiety, depression, and aggressivity. Serotonin is also of importance for basic cell functions such as proliferation, differentiation, maturation, and migration. Serotonin is present in serotonergic neurones in the central nervous system, and in the periphery, it is released from platelets and mast cells (in rodents) after tissue injury.

Pathways for the biosynthesis and biodegradation of serotonin have been characterized in human and rodent skin and in their major cellular populations.^13^ Serotonin in the skin causes pro-oedema, vasodilatory, proinflammatory, and pruritogenic effects via its receptors.^14^

Serotonin generally exerts its effects through seven families of receptors.^15^ Of these subtypes, the 5HT1A and 5HT2A receptors have been suggested to have opposing functions in a variety of cellular and behavioural processes.^16^ The serotonin transporter protein (SERT) regulates 5-HT concentrations in the synaptic cleft via recycling released serotonin, thus terminating the action of 5-HT, and is a target for serotonin reuptake inhibitors.

Psychological stress can provoke many cutaneous dermatoses associated with abnormal epidermal barrier function, such as AD.^17^ Stress involves different neuromediators, such as sensory neuropeptides, including the substance P-NK-1 receptor system, 11 and the serotonergic system, in, for example, raphe.^18^ There is a colocalization of serotonin and sensory neuropeptides in the nervous system.^19^

In the present study, we investigated patients with AD, with histories of chronic stress, taking biopsies for immunohistochemistry, studying nerve density, expression of sensory neuropeptides and serotonin and its receptors, as well as SERT. We investigated levels of salivary cortisol as an indicator of chronic stress.^20^

## Patients and methods

### Patients

Eleven patients (three male and eight female) with a mean age of 34.0 (range, 27–41), who had active AD, moderate to severe (as defined by the criteria by Hanifin and Rajka), 21 were referred to the Neurocutaneous reception at our department by other dermatologists. Each had a history of eczema that worsened during stress.

Ethical permission was obtained from the local ethical committee.

### Salivary cortisol tests

Salivary cortisol samples were obtained from all patients. The salivary samples were collected in plastic vials at 08:00 h on three consecutive days. At 10:00 h p.m. on the last day, 0.25 mg dexamethasone was administered orally, followed by a new cortisol test on the following morning. The samples were stored at –20 °C until analysis. The cortisol concentrations were determined using a radioimmunoassay kit (Spectria Cortisol, Orion Diagnostica, Espoo, Finland) after centrifugation of the samples. The ratio of the mean of the previous three values to the last cortisol value was determined, a low ratio being an indicator of chronic stress.^20^

### Skin biopsies

Four-millimetre biopsies were taken from the lesional skin on the thighs who had marked dryness, grouped papulovesicles, confluent read oedematous areas, and non-lesional skin on the gluteal region on the same patient, after an injection of lidocaine. These sites had not been treated with topical steroids for at least 14 days. The biopsies were fixed in Lanas fix (10% formalin and 0.4% picric acid) at 4 °C for 2 h. They were then rinsed in cold phosphate buffer with 10% sucrose (4 °C) for at least 48 h, snap frozen, and stored at –70 °C until being further processed.

### Immunohistochemistry

Fourteen-micrometre cryostat sections were made on a Dittes cryostate. The slides were incubated overnight (4 °C) in a humid chamber with primary antibodies (Table 1).

**Table 1:** Antibody description

Anti-PGP 9.5, polyclonal, rabbit (1:10 000), UltraClone, Isle of Wight, UK
Anti-5HT1AR 170IV, polyclonal, rabbit (1:5000)^37^
Anti-5HT2AR, monoclonal, mouse (1:500), PharMingen, San Diego, CA, USA^38^
Anti-substance P, polyclonal, rabbit (1:10 000), Bachem, St. Helens, UK
Anti-CGRP, polyclonal, rabbit (1:10 000), Bachem
Anti-CD3, monoclonal, mouse, FITC-conjugated (1:20), PharMingen
SERT, ST51-1, monoclonal, mouse (1:20 000), MabTechnologies, Stone Mountain, GA, USA
Anti-tryptase, monoclonal, mouse (1:5000), Chemicon, Temecula, CA, USA
Biotinylated anti-rabbit, goat (1:200), Vector, Burlingame, CA, USA
Biotinylated anti-mouse, horse (1:200), Vector
Anti-rabbit, swine, FITC-conjugated (1:40), DakoCytomation, Glostrup, Denmark
Streptavidin-conjugated Cy2 (1:2000), Amersham, Buckinghamshire, UK
Streptavidin-conjugated Texas red (1:2000), Vector

The sections were rinsed in PBS and incubated with a biotinylated goat anti-rabbit secondary antibody, in case of polyclonal antibodies, or, in case of the monoclonal antibodies, with a biotinylated horse anti-mouse secondary antibody, for 40 min (Table 1). The primary antibodies were visualized by incubating the sections with the fluorochrome Cy2.

As a control, the primary antibodies (PGP 9.5) were omitted, or the antisera (substance P, CGRP, and 5-HT1A) R were pre-adsorbed with the pure neuropeptides (Bachem) at 10^−5^ mol/L overnight, or with the 5-HT1AR antigenic peptide (synthesized by Ross-Petersen AS, Horsholm, Denmark and used at a concentration of 0.1 mg/mL), respectively, at 4 °C, when no immunoreactivity was obtained. In case of the monoclonal antibodies, control with mouse IgG of the same isotype (DakoCytomation), and in the same dilution as this antibody, was used. In addition, these control experiments resulted in omitted immunoreactivity.

In the double-staining experiments, the same principle method was used, however, with different fluorochromes. Double staining was done for 5-HT2AR or SERT and FITC-conjugated CD3. In these experiments, streptavidin-conjugated Texas red was used to detect the primary antibodies. In the case of SERT, we also used a double staining using a polyclonal antibody to tryptase (from Prof Ilkka Harvima, Department of Dermatology, University of Tampere, Finland).

The sections were then rinsed in PBS, mounted in gelatine/glycerol, and examined with epifluorescence using a Nikon epifluorescence microscope (Eclipse E800, Yokohama, Japan). The Cy2 and FITC-fluorescent structures were visualized with a filter cube with excitation at 465 to 495 nm, whereas Texas red fluorescent elements were seen with a filter cube with excitation at 540 to 580 nm.

Photographs were taken using a video camera system (Nikon digital camera DXM 1200) attached to a fluorescent microscope and connected to a PC computer. The slides were coded before an examination was done by one observer (HR) who was not aware of the results of stress nor location of the biopsies.

Epidermal degree of thickness, acanthosis, was evaluated using a semiquantitative scale. The acanthosis was graded as normal (low), moderate, or high, giving a score of 1, 2, or 3, respectively.

An epidermal staining was evaluated by giving fraction/ratio of the total epidermis.

Labelled nerves and cells were counted in the whole epidermis and papillary dermis. In each biopsy, counts were made of two sections and another two sections 70 µm from the first one.

### Statistical analysis

The Wilcoxon test was used to analyse dependent data and statistical comparisons to test differences between involved and non-involved skin. The Spearman rank correlation coefficient was used to test dependence between the cortisol ratios and number of PGP 9.5-, substance P-, and CGRP-positive nerve fibres and number of 5HT1AR, 5-HT2AR, SERT-positive cells and mast cells, respectively, in the involved and non-involved skin.

The level of significance was set at *P* < 0.05.

## Results

### Cortisol ratio

Cortisol values were obtained from eight patients (mean, 2.9 ± 2.5; Tables 2 and 3). There was a strong tendency (*P* = 0.08) to a correlation between lower serum cortisol ratios and number of 5-HT2AR-positive cells in the papillary dermis of involved skin.

**Table 2:** Non-involved skin

			PGP 9.5 fibres		Substance P fibres		CGRP		5HT1AR		5HT2AR	SERT		
									
Patients	Cortisol	Acanthosis	Epidermis	Papillary dermis	Epidermis	Papillary dermis	Epidermis	Papillary dermis	Epidermal fraction	Papillary dermis cells	Papillary dermis cells	Epidermal fraction	Papillary dermis cells	Mast cells
1	0.6	1	82	117	1	9	2	3	0.2	93	2	0.3	59	113
2	2.3	1	93	113	1	9	1	8	0.2	57	2	0.3	39	52
3	2.4	1	45	113	1	19	0	10	0.3	62	0	0.3	69	62
4	2.4	2	52	130	1	4	0	0	0.3	88	0	0.3	30	142
5	4.1	1	66	109	1	16	2	15	0.3	76	0	0.3	122	169
6	5.1	1	105	165	1	26	1	13	0.3	75	2	0.3	81	107
7	8.1	1	60	102	2	4	1	3	0.3	76	1	0.5	20	320
8	–	1	64	105	1	10	1	9	0.3	84	3	0.3	36	81
9	–	2	97	175	2	14	0	12	1	82	0	0.3	71	90
10	0.5	2	91	130	1	4	1	3	0.3	60	1	0.3	122	43
11	–	1	56	121	1	3	0	1	0.3	57	0	0.5	23	133

**Table 3:** Involved skin

			PGP 9.5 fibers		Substance P fibers		CGRP		5HT1AR		5HT2AR	SERT		
									
Patient	Cortisol	Acanthosis	Epidermis	Pap. derm	Epidermis	Pap. derm	Epidermis	Pap. derm	Epidermal fraction	Pap. derm cells	Pap.derm cells	Epidermal fraction	Pap.derm. cells	Mast cells
1	0.6	3	9	62	0	1	1	1	0.7	118	51	0.5	141	498
2	0.3	3	37	118	17	30	11	28	0.3	51	5	1.0	156	86
3	2.4	2.5	14	93	5	19	1	7	0.5	27	55	0.3	95	112
4	2.4	2	16	53	1	7	0	4	0.2	74	625	0.1	75	176
5	4.1	2	20	63	0	6	1	11	0.5	93	12	0.5	120	200
6	5.1	2	32	82	4	22	0	11	0.5	61	2	0.5	108	140
7	8.1	2	15	35	1	4	0	1	0.5	41	1	0.5	127	628
8	–	1.5	18	82	1	9	1	4	0.5	69	3	0.5	111	204
9	–	3	41	80	8	28	3	5	0.5	27	5	0.5	111	116
10	0.5	2	37	89	5	16	0	1	1	82	238	0.3	45	66
11	–	3	16	76	1	6	0	1	0.5	69	10	0.5	56	216

### Acanthosis

All subjects exhibited a higher degree (*P* < 0.001) of acanthosis in involved [2.4 ± 0.5 (SD)] compared with non-involved (1.3 ± 0.4) skin. The involved skin also showed signs for a light to moderate inflammation and hyperkeratosis.

### PGP 9.5

**fig. 1:**
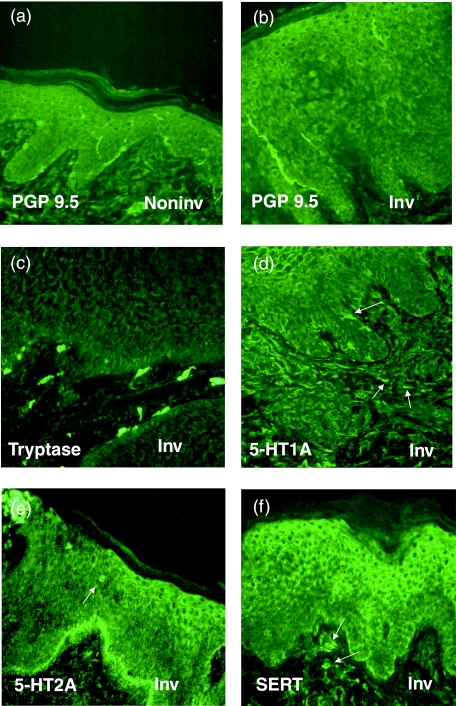
Epidermal PGP 9.5-positive nerve fibres in (a) non-involved (b) and involved AD skin. Tryptase-positive mast cells in proximity to the basal membrane (c) and 5-HT1AR expression in involved skin (d). Note the apical epidermal expression of 5-HT1AR, the dendritic melanocytes (*arrow*), as well as the mononuclear cells (*arrows*) in the dermis. 5-HT2AR–positive apical epidermis, basal membrane, and cells (*arrow*) that also intrude into the epidermis of involved skin (e). SERT expression in the epidermis and dermal cells (*arrow*) of involved skin (f). Magnification, ×200.

**fig. 2:**
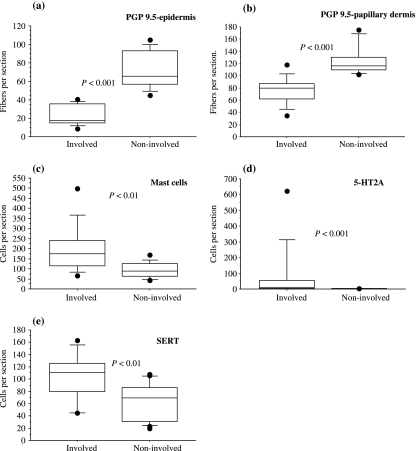
Graphs showing number of epidermal (a) and dermal (b) PGP 9.5-positive fibres, mast cells (c), 5-HT2AR- (d), and SERT- (e) positive cells in involved and non-involved skin, respectively.

There was a decrease (*P* < 0.001) in the number of PGP 9.5–positive fibres (fig. 1a,b, shown for epidermis), in involved skin (22.9 ± 10.8) and 75.5 ± 21.2, fibres per section in the epidermis and dermis, respectively, compared with the non-involved skin (73.8 ± 19.7 and 125.3 ± 22.1; fig. 2a,b).

### Mast cells

There was an increase (*P* < 0.01) in mast cells, round to dendritic in the involved skin, 222.0 ± 169.7 cells per section, compared with non-involved skin (119.3 ± 73.6; fig. 2c). The mast cells in the involved skin showed a tendency to attach to the epidermis (fig. 1c).

### Sensory neuropeptides

In the epidermis, 3.8 ± 5.0 fibres per section were positive for substance P (data not shown) in the involved skin, compared with 1.1 ± 0.4 in the non-involved skin. In the papillary dermis, there was no significant distinction between involved (13.3 ± 9.7) and non-involved (10.2 ± 0.6) skin.

There were also a few fibres positive for CGRP in the epidermis (data not shown) but no significant difference in fibre density between involved (1.6 ± 3.0) and non-involved (0.8 ± 0.7) skin, as was the case for the papillary dermis (involved skin, 6.6 ± 7.6 and non-involved skin, 6.9 ± 5.1).

In one of the patients with a low cortisol ratio, there was a large number of substance P*-* and CGRP-positive fibres in both the epidermis and dermis.

### Serotonin receptor 1A

The staining of the epidermis for 5-HT1AR (fig. 1d) was more extensive (*P* = 0.05) in the involved compared with the non-involved skin. The outer half part, 0.5 ± 0.2 of total epidermal layer, was positively stained compared with one third, 0.3 ± 0.2, in the non-involved epidermis. 5-HT1AR–positive melanocyte-like cells with varying dendricity were seen on the basal membrane; their dendrites seemed longer in the involved skin.

There was no difference between the number of 5-HT1AR–positive cells in the papillary dermis in the involved (72.9 ± 31.2 cells per section) and non-involved (74.1 ± 12.1) skin. Double staining showed that the majority of the 5-HT1AR-positive cells in papillary dermis were positive for tryptase (not shown).

### Serotonin receptor 2A

The apical epidermis was stained up to half of its thickness in 5 of 11 patients in the involved skin and in 6 of 11 patients in the non-involved skin (fig. 1e). There was a staining of the basal membrane, which often extended to the basal cell layer in the involved skin in 6 of 11 patients and in the non-involved skin in 5 of 11 patients.

**fig. 3:**
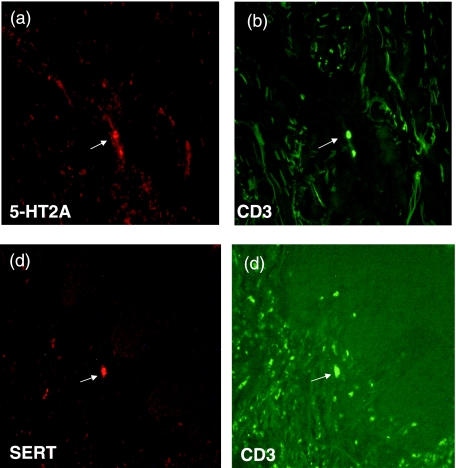
Double staining for 5-HT2AR (a) and CD3 (b), SERT (c), and CD3 (d) respectively in the papillary dermis of involved skin. a and c, Texas red; b and d, FITC. Magnification, ×200.

5-HT2AR-positive cells were found in involved skin both in the epidermal and the dermal layers. An increased (*P* < 0.001) number of 5-HT2AR-positive cells was found in the papillary dermis of the involved skin (95.4 ± 184.5 cells per section) compared with non-involved skin (0.7 ± 0.8; fig. 2d). Double staining showed that these 5-HT2AR-positive cells were positive for CD3 (fig. 3a,b).

### SERT

There was an increased (*P* < 0.05) immunoreactivity in the upper part of the epidermis (fig. 1f**)**, a higher fraction of the epidermis (0.5 ± 0.2) being affected in the involved compared to the non-involved (0.4 ± 0.1) skin. An increased (*P* < 0.01) number of SERT-positive cells were found in the dermis of the involved (104.1 ± 32.6) compared with non-involved (61.1 ± 34.6) skin (fig. 2e). Some patients also had SERT-positive cells in the epidermis of the involved skin. The majority of the SERT-positive cells were CD3 positive (fig. 3c,d), whereas a few were tryptase positive (data not shown).

## Discussion

In the present investigation, we report a decreased innervation in the involved compared to non-involved skin in AD patients. We found an increased epidermal fractional immunoreactivity for 5-HT1AR and SERT and an increased number of dermal 5-HT2AR and SERT-positive cells, also the number of mast cells being increased in the involved skin.

It has earlier been reported that the density of cutaneous nerves is higher in atopic skin than in the skin of healthy controls.^22^, ^23^ The quality of nerve fibres are also changed in AD patients, the nerve endings being thin and running straight through the epidermis.^24^

The free nerve endings in lesional skin of AD might be in an active state of excitation that have effect on keratinocytes.^25^

In a previous study, 26 there was a decreased number of PGP 9.5–positive fibres and NGF receptor in the epidermis in patients with prurigo nodularis. Maybe the loss of fibres is due to mechanical damage, which might lead to collateral sprouting of other nerve fibres or regeneration of the damaged nerve fibres.

There was an increase in the ratio of 5-HT1AR and SERT fractions of the immunoreactive epidermis in involved compared to non-involved skin. This is interesting since there is a relation between 5-HT1AR and SERT, the function of SERT and influence on the 5-HT concentration being associated with a modulation of 5-HT1A autoreceptors.^27^

A modulation of the skin barrier has been reported to be due to psychological stress.^17^

The stress response in the skin seems to be served by locally expressed neuroendocrine activities.^28^ The skin is a powerful steroidogenic tissue and involved in the production of steroids.^29^

The creation of a skin barrier is linked to a decreased production of lamellar bodies, which in turn is attributed to a diminished *de novo* synthesis of epidermal lipids, including cholesterol.^17^ Cholesterol is an essential component of eucaryotic membranes. It is of special interest that the function of the 5-HT1AR is dependent on cholesterol (see ref. 30). In this respect, it may be mentioned that cholesterol can also be metabolized to steroid hormones in the skin.^31^ In addition, the 5-HT1A and 5-HT2A receptor functions have been reported to be dependent on steroids.^32^

Regarding the intrusion of 5-HT2AR-positive cells into the epidermis, this might be due to effect on interstitial collagenases, being mediated by the 5-HT2AR (see ref. 33). An antagonist to the 5-HT2A receptor has been shown to decrease the expression of matrix metalloproteinase.^34^

There was a strong tendency (*P* = 0.08) to a correlation between lower serum cortisol ratios, being an indicator of chronic stress, and number of 5-HT2AR-positive cells in the papillary dermis of the eczematous skin. The 5-HT2AR in the brain cortex has been reported to be up-regulated during chronic stress due to maternal deprivation.^35^

The mast cells in the present investigation could be seen to be increased in the involved compared to non-involved skin and as well to attach to the basement membrane. Mast cells are known to be able to activate matrix metalloproteinases via the production of tryptase.^36^ Tryptase itself and the activated matrix proteinases can degrade various components of the pericellular/extracellular matrix.

It is of value for the AD patients to reduce stress in addition to the use of topical or oral treatment for the inflammation. In addition, pharmacological treatment targeting the serotonergic receptors and SERT might be a possible treatment strategy.
